# Do North Atlantic eels show parallel patterns of spatially varying selection?

**DOI:** 10.1186/1471-2148-14-138

**Published:** 2014-06-20

**Authors:** Malene G Ulrik, José Martín Pujolar, Anne-Laure Ferchaud, Magnus W Jacobsen, Thomas D Als, Pierre Alexandre Gagnaire, Jane Frydenberg, Peder K Bøcher, Bjarni Jónsson, Louis Bernatchez, Michael M Hansen

**Affiliations:** 1 Department of Bioscience, Aarhus University, Ny Munkegade 114, Bldg. 1540, DK-8000 Aarhus C, Denmark; 2 National Institute of Aquatic Resources, Technical University of Denmark, Vejlsøvej 39, DK-8600 Silkeborg, Denmark; 3 Department of Biomedicine-Human Genetics, Aarhus University, DK-8000 Aarhus C, Denmark; 4 ISEM (Institut des Sciences de l’Evolution Montpellier), Université Montpellier II, 34095 Montpellier, France; 5 Biopol, Marine Biology and Biotechnology Center, Einbúastígur 2, IS545 Skagastrond, Iceland; 6 IBIS (Institut de Biologie Intégrative et des Systèmes), Université Laval, G1V 0A6 Québec, Canada

**Keywords:** Adaptation, European eel, Genetic-by-environment associations, Parallel selection, Single nucleotide polymorphisms

## Abstract

**Background:**

The two North Atlantic eel species, the European and the American eel, represent an ideal system in which to study parallel selection patterns due to their sister species status and the presence of ongoing gene flow. A panel of 80 coding-gene SNPs previously analyzed in American eel was used to genotype European eel individuals (glass eels) from 8 sampling locations across the species distribution. We tested for single-generation signatures of spatially varying selection in European eel by searching for elevated genetic differentiation using F_ST_-based outlier tests and by testing for significant associations between allele frequencies and environmental variables.

**Results:**

We found signatures of possible selection at a total of 11 coding-gene SNPs. Candidate genes for local selection constituted mainly genes with a major role in metabolism as well as defense genes. Contrary to what has been found for American eel, only 2 SNPs in our study correlated with differences in temperature, which suggests that other explanatory variables may play a role. None of the genes found to be associated with explanatory variables in European eel showed any correlations with environmental factors in the previous study in American eel.

**Conclusions:**

The different signatures of selection between species could be due to distinct selective pressures associated with the much longer larval migration for European eel relative to American eel. The lack of parallel selection in North Atlantic eels could also be due to most phenotypic traits being polygenic, thus reducing the likelihood of selection acting on the same genes in both species.

## Background

Parallel adaptive changes under replicated environmental conditions have been particularly valuable for understanding evolutionary processes in natural populations. One of the classical questions in evolutionary biology concerns whether different species and populations within species will adapt to the same agent of selection in the same way or whether the response will involve different traits and genes [1,2]. Parallel genotypic adaptation appears to be frequent and occurs at all taxonomic levels from microbes and plants to humans [3,4] and is likely to result in changes at a relatively small number of genes [5]. For instance, the study of Colosimo et al. [6] demonstrated that selection on a single gene, ectodysplasin (*Eda*), is responsible for the parallel reduction of armor plates in freshwater populations of threespine stickleback *Gasterosteus aculeatus*. However, more complex physiological processes relevant in the context of parallel freshwater adaptation of threespine sticklebacks are influenced by several genes, each of small effect [7-10]. Using a survey of the published literature on parallel adaptation of independent lineages of natural populations, Conte et al. [5] concluded that divergence at loci under selection is most likely to be based on standing genetic variation derived from a common ancestor rather than mutations occurring *de novo* after divergence. Hence, probability of gene reuse is plausibly higher in closely related species, which are likely to show similar divergence at loci subjected to similar selection pressures [11].

An excellent opportunity to test for genetic parallelism exists in the two North Atlantic eel species, the European eel (*Anguilla anguilla*) and the American eel (*A. rostrata*). Both species are morphologically almost indistinguishable, with the number of vertebrae being regarded as the best diagnostic character between species [12]. Divergence time between the two species remains largely unresolved, encompassing between 1.5 and 5.8 million years [13-15]. Remarkably, although mitochondrial DNA lineages of the two species are reciprocally monophyletic [13], differentiation at nuclear loci is surprisingly low (F_ST_ = 0.055 [16]; F_ST_ = 0.018 [17]; F_ST_ = 0.06 [18]; F_ST_ = 0.09 [19]), suggestive of ongoing gene flow. In this sense, it is well established that the spawning grounds of the two species overlap in the Sargasso Sea and there is also overlap in spawning time [20]. European and American eels are known to hybridize, with hybrids observed almost exclusively in Iceland [21-23]. Hence, the sister species status of European and American eel and the low but biologically significant gene flow makes them an adequate system in which to test the occurrence of selection at homologous loci within each species.

North Atlantic eels have a catadromous life cycle and after spawning in the Sargasso Sea, larvae are transported by the Gulf Stream and other currents to the shores of North America and Europe/North Africa, respectively. Upon reaching the continental shelf, larvae metamorphose into glass eels, which complete the migration into riverine, estuarine and coastal feeding habitats and grow up as yellow eels. After a highly variable feeding stage, yellow eels metamorphose into partially mature silver eels that migrate back to the Sargasso undertaking a journey of about 2,000 km for the American eel and 5,000-6,000 km for the European eel. Upon arriving in the Sargasso Sea, eels reproduce and die [24]. During the continental phase, eels occupy a broad range of habitats from the Caribbean to Greenland in the western Atlantic (American eel) and from Morocco to Iceland in the eastern Atlantic (European eel). The presence of eels in extremely heterogenous environments in terms of temperature (i.e. from subtropical to subarctic), salinity (i.e. from freshwater to marine), substrate, depth or productivity along their geographic distribution makes them ideal species in which to study the consequences of spatially varying selective pressures that often result in local adaptation of ecologically important traits [1,25,26]. Beginning with Levene [27], who introduced the first theoretical model for examining the impact of diversifying selection in space, a number of studies have shown that balancing selection due to spatial heterogeneity is an important mechanism responsible for the maintenance of genetic polymorphism (reviewed in [28]). Genetic variation in a spatially heterogenous environment may be maintained even when dispersal results in complete mixing of the gene pool [1]. However, under such a panmixia scenario, in which offspring are distributed to environments at random independently of the environment experienced by the parents, local selection cannot to lead to local adaptation [29]. In the case of eels, owing to panmixia in both European [19,30] and American eel [31] and random larval dispersal across habitats, heritable trans-generational local adaptation is not possible although single-generation footprints of selection can still be detected. In this sense, significant geographic clines at allozyme loci have been detected in both European [32] and American eel [33]. In the most comprehensive study to date, Gagnaire et al. [26] found evidence for spatially varying selection at 13 coding genes in American eel showing significant correlations between allele frequencies and environmental variables (latitude, longitude and temperature) across the entire species range.

In this study, we tested for single-generation signatures of spatial varying selection in European eel and compared the results to those obtained by Gagnaire et al. [26]. We genotyped glass eels from 8 sampling locations across the geographic distribution of the species, using the same set of SNPs analyzed by Gagnaire et al. [26] in American eel. We used two main analytical approaches, one that identifies outliers as those markers with greater differentiation among all SNPs and a second based on determining positive associations between allele frequencies and environmental factors. Following the positive associations observed by Gagnaire et al. [26] in American eel, variables used in our study were degrees North latitude, degrees East/West longitude and sea-surface temperature at river mouth. We specifically wanted to test whether the same genes were under spatially varying selection in both European and American eel, hence providing evidence for parallel patterns of local selection, or whether the response involved a different set of genes. Considering their sister species status and the existence of gene flow between species, together with the similar environmental conditions they encounter [34], we hypothesize that the two North Atlantic eel species show parallel patterns of selection at the same loci.

## Results

Genetic diversity values for all genotyped individuals at 80 SNPs are summarized in Table 1. 17 out of 80 loci were monomorphic and 63 were polymorphic in European eel, although frequency of the most common allele was >0.95 at 27 loci. Diversity indices were higher in American eel (H_o_ = 0.302; H_e_ = 0.306; P_95_ = 0.896; P_99_ = 0.922) than in European eel (H_o_ = 0.149; H_e_ = 0.157; P_95_ = 0.429; P_99_ = 0.610), suggestive of a strong ascertainment bias effect due to the fact that SNPs were identified in American eel.

**Table 1:** **Details of all genes and loci studied, including observed (H**_**o**_**) and expected (H**_**e**_**) heterozygosities at all loci in American (AR) and European eel (AA)**

**Locus**	**Gene**	**AR**		**AA**	
		**H**_**o**_	**H**_**e**_	**H**_**o**_	**H**_**e**_
40S_S18_1401	40s ribosomal protein s18	0.389	0.375	0.344	0.312
60S_L10A_21874	60s ribosomal protein L10a	0.250	0.219	0.282	0.306
ACT_A3B_8646	Actinin alpha 3b	0.300	0.255	0.006	0.006
ACTB_21752	Beta-actin	0.474	0.411	0.213	0.244
ACYL_13914	Acyl carrier protein	0.421	0.388	0.320	0.310
ADH_3	Alcohol dehydrogenase class-3	0.263	0.361	0.229	0.351
ADSS_L1_15447	Adenylosuccinate synthetase isozyme 1	0.158	0.229	0.016	0.016
ALD_R	Aldose reductase	1.000	0.500	0.395	0.345
ALDH_2_16634	Aldehyde dehydrogenase 2	0.947	0.499	0.468	0.428
ANK_R_13478	Ankyrin repeat domain-comtaining protein 1	0.250	0.289	0.000	0.000
ANN_A11_16176	Annexin A11	0.200	0.180	0.171	0.172
ANX_2_249	Annexin A2-A	0.100	0.375	0.016	0.034
ARF_4_18099	ADP-ribsylation factor 4	0.444	0.346	0.019	0.019
ATP_BC_259	ATP-bindincasette sub-family A member 1	0.450	0.439	0.022	0.022
BPNT_1_18778	3′(5′),5′-biphosphate nucleotidase 1	0.263	0.411	0.025	0.025
CLIC_5_10148	Chloride intracellular channel 5	0.250	0.219	0.521	0.492
COI_17591	Cytochrome oxidase subunit I	0.000	0.000	0.010	0.009
COP_9_18132	266S protease regulatory subunit 7	0.300	0.255	0.035	0.034
CSDE_1_11069	Cold shock domain-containing protein E1	0.316	0.266	0.019	0.019
CSDE_1_19713	Cold shock domain-containing protein E1	0.474	0.411	0.066	0.064
CST_21113	Cystatin precursor	0.421	0.499	0.379	0.393
CYT_BC1_9061	Cytochrome b-c1 complex subunit 2	0.200	0.180	0.000	0.000
EF_1G_4796	Translation elongation factor 1 gamma	0.400	0.320	0.000	0.000
EF2_10494	Translation elongation factor 2	0.200	0.180	0.000	0.000
EIF_3F_341	Translation elongation factor 3 subunit F	0.211	0.332	0.113	0.146
EIF_3J_11587	Translation elongation factor 3 subunit J	0.300	0.375	0.079	0.082
FER_H_20955	Ferritin heavy subunit	0.421	0.432	0.009	0.009
FGB_47	Fibrinogen Beta Chain	0.300	0.255	0.000	0.000
GAPDH_20355	Glyceraldehyde-3-phoshpate dehydrogenase	0.200	0.180	0.222	0.197
GDE1_2508	Glycerophosphochlorine phosphodiesterase	0.389	0.313	0.000	0.000
GOG_B1_15792	Golgin sub-family B member 1	0.150	0.289	0.050	0.049
GPX_4_19607	Glutathione peroxidase 4	0.100	0.095	0.000	0.000
HMG_T_9973	High mobility group-T protein	0.050	0.049	0.025	0.025
HSP_90A_15666	Heat shock protein 90 alpha	0.158	0.229	0.063	0.061
HSP_90B_21100	Heat shock protein 90 beta	0.150	0.139	0.107	0.107
HSPE_1_17854	10 kDa heat shock protein	0.368	0.362	0.009	0.009
IF_RF2_19747	Interferon regulatory factor 2	0.150	0.139	0.000	0.000
JAM_3_13916	Junctional adhesion molecule 3b	0.053	0.051	0.131	0.165
KRT_13_20618	Keratin	0.350	0.499	0.325	0.317
KRT_A_15738	Keratin alpha-like	0.000	0.000	0.022	0.022
LBL_L2_20921	No hit	0.000	0.000	0.000	0.000
LDH_B_9441	Lactase dehydrogenase B	0.600	0.455	0.025	0.025
MDH_1393	Malate dehydrogenase	0.263	0.450	0.298	0.493
MYH_14857	Superfast myosin heavy chain	0.300	0.255	0.563	0.496
NADH_4_21742	NADH dehydrogenase subunit 4	0.000	0.0180	0.000	0.000
NADH_5_17101	NADH dehydrogenase subunit 5	0.000	0.255	0.000	0.019
NADH1_10_21119	NADH dehydrogenase 1 alpha subunit 10	0.300	0.455	0.000	0.000
NCP_2_15547	Nucleolar complex protein 2	0.350	0.289	0.085	0.087
NEX_19953	Nexilin	0.450	0.489	0.476	0.496
NGD_21138	Neuroguidin	0.450	0.469	0.238	0.273
NRAP_1541	Nebulin-related anchoring protein	0.500	0.455	0.031	0.031
PA2G4_2600	Proliferation associated protein 2G4	0.444	0.401	0.442	0.481
PFN_15113	Profilin-2	0.400	0.375	0.000	0.000
PGD_18096	6-phosphogluconate dehydrogenase	0.000	0.000	0.042	0.053
PGI_1	Phosphoglucose isomerase-1	0.211	0.188	0.338	0.348
PGI_2	Phosphoglucose isomerase-2	0.368	0.478	0.456	0.498
PGK_1_11454	Phosphoglycerate kinase 1	0.474	0.362	0.090	0.092
PRP_40_16504	Pre-mRNA-processing factor 40 homolog A	0.150	0.219	0.205	0.214
PSA_4_21534	Proteasome subunit alpha type-4	0.350	0.439	0.484	0.498
PSME_1_21196	Proteasome activator	0.235	0.458	0.441	0.457
RFC_3_18186	Replication factor C subunit 3	0.350	0.289	0.416	0.368
RTF_1_21288	RNA polymerase-associated protein RTF1 homolog	0.632	0.432	0.339	0.374
SDH_O	Sorbitol dehydrogenase	0.000	0.000	0.000	0.000
SLC_25A5_19808	ADP/ATP translocase 2	0.450	0.499	0.006	0.006
SM_22_6449	Transgelin	0.550	0.499	0.074	0.083
SN4_TDR_374	Taphylococcal nuclease domain-containing protein 1	0.526	0.465	0.182	0.191
TENT_02_11046	No hit	0.111	0.105	0.318	0.362
TENT_03_12589	Collagen type XXVIII alpha 1 a	0.150	0.219	0.041	0.040
TENT_05_19704	No hit	0.500	0.461	0.013	0.013
TENT_06_16512	Protein Phosphatase regulatory subunit	0.105	0.266	0.510	0.484
TENT_07_21161	No hit	0.700	0.451	0.744	0.477
TNNT_2E_20968	Troponin T2e	0.000	0.000	0.168	0.196
TRIM_35_8416	Tripartite motif-contaning protein 35	0.368	0.450	0.361	0.426
TTN_B_20952	Titin b	0.421	0.332	0.003	0.003
TUB_A_19211	Tubulin alpha 2	0.550	0.489	0.010	0.009
UBI_A52_5049	Ubiquitin A-52 residue ribosomal protein fusion product 1	0.474	0.362	0.058	0.056
UGP_2_2128	UDP-glucose pyrophosphorylase 2	0.316	0.808	0.236	0.771
UGP_A_2307	Glycerol-3-phosphate transporter subunit	0.600	0.334	0.733	0.466
UNA_SINE2_16912	Eel Short interspersed elements	0.000	0.000	0.000	0.000
ZETA_15177	Tyr 3-monooxygenase/Trp 5-monooxygenase activation protein	0.421	0.388	0.246	0.245

Three loci deviated significantly from Hardy-Weinberg expectations after Bonferroni correction: locus UGP_2_2128, showing a deficit of heterozygotes, and loci TENT_0721161 and UGP_A_2307, showing an excess of heterozygotes. However, those loci were not excluded from the analysis as they could reflect selection.

Two loci (ALD_R and PSME_1_21196) were genotyped at all locations except Tiber, so all tests for selection were conducted considering all 8 locations and 78 loci (excluding ALD_R and PSME_1_21196) and on a restricted data set with 7 locations (excluding Tiber) and 80 loci.

**Figure 1:**
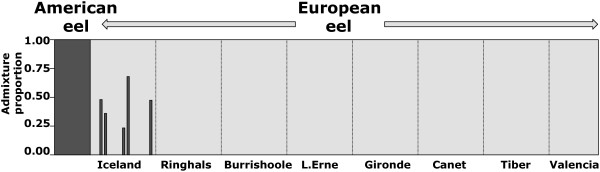
**Admixture analysis using STRUCTURE.** Individuals were assigned assuming the presence of two groups (K = 2). Each vertical line represents one individual, partitioned into segments according to the proportion of European eel (light) and American eel (dark).

Overall genetic differentiation was low (F_ST_ = 0.0079). Using STRUCTURE, a scenario with two clusters (K = 2) corresponding to the two species was the most likely, with no substructuring within species (Figure 1). In the same analysis, a total of 5 individuals from Iceland were identified as admixed individuals with 90% probability intervals that did not overlap with zero. While the SNPs used in this study were not species-diagnostic, results were concordant with the study of Pujolar et al. [23], in which the same individuals were identified as admixed on the basis of 86 species-diagnostic SNPs, encompassing four F1 hybrids and one second generation backcross. Hybrids were only observed in Iceland and were absent in the remaining European locations. All hybrid individuals were removed from further analyses.

The selection detection workbench LOSITAN identified three outlier loci possibly under diversifying selection using the full data set, GAPDH_20355, MYH_14857 and ALDH_2_16634, with p < 0.05 (Table 2). When using the restricted data set with 7 locations, a further outlier was also identified, ALD_R (p = 0.000). Using the complete data set with 8 locations BAYESCAN identified a single outlier, GAPDH_20355, showing a high F_ST_ value of 0.123 relative to the background F_ST_ (Table 2). At this locus, the alpha coefficient was positive, suggestive of diversifying selection. When using the restricted data set with 7 locations, ALD_R was also identified as outlier in addition to GAPDH_20355, showing a high F_ST_ value of 0.091 and a positive alpha coefficient.

**Table 2:** **Detection of outlier loci using the F**_**ST**_**-outlier approach implemented in LOSITAN and BAYESCAN based on the full data set with 8 locations (78 loci) and a reduced data set with 7 locations (80 loci)**

**LOSITAN**					
**Locus**	**Gene**	**Het**		**F**_**ST**_	**p value**
**8 populations**					
MYH_14857	Superfast myosin heavy chain	0.490		0.042	0.018
GAPDH_20355	Glyceraldehyde-3-phosphate dehydrogenase	0.213		0.246	0.000
ALDH_2_16634	Aldehyde dehydrogenase 2	0.428		0.032	0.047
**7 populations**					
GAPDH_20355	Glyceraldehyde-3-phosphate dehydrogenase	0.231		0.256	0.000
ALDH_2_16634	Aldehyde dehydrogenase 2	0.435		0.036	0.012
ALD_R	Aldose reductase	0.346		0.156	0.000
**BAYESCAN**					
**Locus**	**Gene**	**BPP**	**q value**	**alpha**	**F**_**ST**_
**8 populations**					
GAPDH_20355	Glyceraldehyde-3-phosphate dehydrogenase	1.000	0.000	3.376	0.123
**7 populations**					
GAPDH_20355	Glyceraldehyde-3-phosphate dehydrogenase	1.000	0.000	2.424	0.116
ALD_R	Aldose reductase	1.000	0.000	2.150	0.091

Heterozygosity and F_ST_ values are detailed for all outlier loci detected in LOSITAN. Bayesian posterior probabilities (BPP), q values, alpha coefficients and F_ST_ values are detailed for all outlier loci detected in BAYESCAN.

A generalized linear model between allelic frequencies and explanatory variables using the full data set revealed significant associations with temperature (2 loci), latitude (3 loci) and longitude (5 loci) (Table 3). One locus (GAPDH_20355) showed a significant association with both latitude and longitude. No interactions with explanatory variables were found at any locus. Locus ALD_R showed a positive association with longitude when using the restricted data set with 7 locations (p = 0.016).

**Table 3:** Statistical associations between allele frequencies and a set of three explanatory variables (TEMP, temperature; LAT, latitude; LON, longitude) assessed using generalized linear models (GLM) and BAYENV based on the full data set with 8 locations (78 loci) and a reduced data set with 7 locations (80 loci)

**GLM**		
**Locus**	**Gene**	**p value**
**8 populations**		
TRIM_35_8416	Tripartite motif-containing protein 35	TEMP (r = 0.78; p = 0.023)
NEX_19953	Nexilin	LAT (r = 0.80; p = 0.018)
GAPDH_20355	Glyceraldehyde-3-phosphate dehydrogenase	LAT (r = 0.72; p = 0.045) + LON (r = 0.74; p = 0.042)
KRT_13_20618	Keratin	TEMP (r = 0.81; p = 0.015)
UBI_A52_5049	Ubiquitin A-52	LON (r = 0.72; p = 0.044)
PGK_1_11454	Phosphoglycerate kinase	LON (r = 0.76; p = 0.031)
PSA_4_21534	Proteasome subunit alpha type-4	LAT (r = 0.80; p = 0.018)
ALDH_2_16634	Aldehyde dehydrogenase 2	LON (r = 0.72; p = 0.044)
CST_21113	Cystatin precursor	LON (r = 0.72 p = 0.043)
**7 populations**		
TRIM_35_8416	Tripartite motif-containing protein 35	TEMP (r = 0.77; p = 0.042)
NEX_19953	Nexilin	LAT (r = 0.76; p = 0.045)
GAPDH_20355	Glyceraldehyde-3-phosphate dehydrogenase	LON (r = 0.73; p = 0.046)
KRT_13_20618	Keratin	TEMP (r = 0.82; p = 0.023)
PSA_4_21534	Proteasome subunit alpha type-4	LAT (r = 0.76; p = 0.047)
ALD_R	Aldose reductase	LON (r = 0.82; p = 0.016)
**BAYENV**		
**Locus**	**Gene**	**BF**
**8 populations**		
GAPDH_20355	Glyceraldehyde-3-phosphate dehydrogenase	LAT (BF = 7.450)
**7 populations**		
GAPDH_20355	Glyceraldehyde-3-phosphate dehydrogenase	LAT (BF = 4.501)
ALD_R	Aldose reductase	LON (BF = 3.295)

Correlation coefficients and p-values are detailed for all loci showing significant associations using GLM. Bayes Factors (BF) are presented for all positive associations in BAYENV.

On the other hand, one positive association was found using BAYENV, locus GAPDH_20355 and latitude, with a Bayes Factor of 7.450 (Table 3), while no associations were found for the rest of loci (Bayes Factor <3). In addition, a positive association was found between locus ALD_R and longitude, with a Bayes Factor of 3.295, when considering the reduced data set with 7 locations.

Overall, we identified a total of 11 candidate loci, 4 from outlier tests plus 7 additional loci from the analysis of association of allelic frequencies with explanatory variables.

## Discussion

### Signatures of local selection in European eel

The observation of a small set of SNPs showing significantly high genetic differentiation in comparison with the background F_ST_ and significant associations between allele frequencies and environmental variables is consistent with the action of spatially varying selection associated with the highly heterogenous habitats that glass eels colonize throughout their geographic range. Our findings fit the general prediction that selective pressures frequently vary in space, often resulting in local selection of ecologically adaptive traits [1].

Overall, we found signatures of selection at a total of 11 loci. We found discordances between the two approaches used (F_ST_ outlier tests vs. environmental-association methods), with few SNPs identified as targets of selection by both methods and with a higher number of candidates identified using a generalized linear model. This is in agreement with recent studies showing that SNPs positively correlated with environmental variables were not F_ST_ outliers [35-37]. It has been suggested that SNP-environment associations are more sensitive to detect subtle clines in allele frequencies than F_ST_ outlier tests and that both approaches might be complementary but not concordant when testing for selection [38].

Nevertheless, three loci in our study (GAPDH, ALDH2 and ALD_R) showed higher genetic differentiation than the background F_ST_ together with significant associations between allele frequencies and environmental variables. All three are genes with major metabolic functions: GAPDH (Glyceraldehide 3-phosphate dehydrogenase) is part of the glycolysis pathway and catalyzes the conversion of glyceraldehyde 3-phosphate to D-glycerate 1,3-bisphosphate; ALDH2 (Aldehyde dehydrogenase 2) belongs to the aldehyde dehydrogenase family of enzymes that catalyze acetaldehyde to acetic acid and is the second enzyme of the major oxidative pathway of alcohol metabolism; ALD_R (Aldose reductase) catalyzes the reduction of glucose to sorbitol, the first step in the polyol pathway of glucose metabolism. Besides these genes, a positive environment correlation was observed for PGK (Phosphoglycerate kinase), which is a transferase enzyme in glycolysis acting in the first ATP-generating step of the glycolytic pathway. Surprisingly, none of the above genes linked to metabolism showed positive associations with temperature, arguably an environmental variable of key importance influencing enzymatic activities and metabolic pathways [39,40]. In eels, decreased metabolic activities have been observed below certain threshold temperatures in both European [41] and American eel [42], and distinct behaviour patterns such as upstream migration of glass eels have been shown to be temperature-related [43]. Since it could be argued that temperature at other time intervals might be more relevant than the 30 day-interval used in our study, we re-conducted a generalized linear model between allelic frequencies at GAPDH, ALDH2 and ALD_R with temperature using other time intervals (10 days, 3 months, 6 months, 12 months). No significant associations were found at any of the locus, which suggests that other agents of selection than temperature could underlie the significant associations found. In the case of aldose reductase, this is an enzyme induced by hyperosmolarity stress [44]. Spatially varying selection in European eel regarding osmoregulation seems plausible, since eels occupy highly variable habitats across Europe in terms of salinity, including both fresh and salt-water (i.e. marine and brackish) habitats [45].

Besides genes involved in metabolic functions, several genes involved in defense response showed a positive environment correlation, including TRIM35 (Tripartite motif-containing protein 35), CST (Cystatin precursor), PSA4 (Proteasome subunit alpha-4) and UBIA52 (Ubiquitin A52), all involved in catalytic activity. Interestingly, TRIM35 is a gene implicated in processes associated with innate immunity [46]. Together with other TRIM family genes, TRIM35 is located on a region of significantly elevated genetic diversity (LG XIII) in the threespine stickleback, which suggests that the polymorphism increase on LG XIII has been likely driven by selection on innate immunity genes [7]. While allele frequencies at TRIM35 were positively correlated with temperature, allele frequencies at CST, UBIA52 and PSA4 were associated with geographic coordinates. As in the case of metabolism, it is possible that other explanatory variables (e.g. productivity, oxygen level, salinity, pollution and parasite load) may play a role in defense response rather than temperature or geographic coordinates.

### No apparent parallel patterns of selection in North Atlantic eels

The contrasting pattern of spatially varying selection observed in European eel (this study) and American eel [26] using the same panel of candidate SNPs suggests no common genetic-by-environment associations between North Atlantic eels. Using generalized linear models, Gagnaire et al. [26] found significant associations with environmental variables at 8 loci within glass eels (ACP, ANX2, GPX4, HSP90A, MDH, NRAP, PRP40 and UGP2), none of which are common with the 10 loci that showed significant associations in our study using the same statistical approach (ALDH, ALD_R, CST, GAPDH, KRT, NEX, PGK, PSA4, TRIM35, UBIA52) and also conducted on glass eels.

However, two loci (TRIM35 and CST) showed some evidence of selection in both species. In American eel, TRIM35 showed the highest F_ST_ value detected between localities (F_ST_ = 0.174) although no correlation with environmental variables was detected at this locus. CST showed signatures of selection within cohorts of juveniles but not within glass eels [26].

While most loci under selection in Gagnaire et al. [26] represented metabolic genes associated with temperature, those genes with a major role in metabolism in our study (GAPDH, ALD_R, ALDH, PGK) did not show a positive association with temperature despite the similar temperature ranges encountered by both species (European eel: 4.2-15.1°C, American eel: 3.4-19.8°C). However, the different signatures of selection between species could be due to distinct selective pressures associated with the much longer larval migration for European eel than for American eel, with estimates ranging from 7 months to 2 years for European eel depending on the assumptions and methods used, whereas estimates for American eel range between 6 and 12 months [47]. The one extra year that possibly European eel larvae spend in the open sea could impose a different set of selective agents relative to American eel.

In contrast with our findings, the recent survey of Conte et al. [5] on published literature of repeated phenotypic evolution in natural populations concluded that the probability of gene reuse was high (on average 55%). However, the survey was based on candidate gene studies, which might have biased upward the reuse estimates. The lack of parallel selection patterns in North Atlantic eels is unanticipated owing to the sister species status of European and American eel and the permeable barrier to gene flow between species. A recent study using a RAD-sequencing approach to identify diagnostic markers between the two species found a small proportion of fixed SNPs (<0.5%), while most of the SNPs showed low non-significant differentiation that suggest that most of the genome is homogenized by gene flow [23]. One possible explanation to the lack of parallel selection is that complex phenotypic traits affected by local selection might have a highly polygenic basis, hence influenced by several genes, each with a small contribution to the ultimate function [48,49]. Parallel selection is more likely to occur when the adaptive response is controlled by a single gene, i.e. the *Eda* gene and armor plate reduction [6,50] and the Kit ligand *Kitlg* gene and pigmentation [51] in threespine sticklebacks or the melonacortin-1 receptor *Mc1r* and colour pattern in beach mice [52]. More complex traits are likely to involve a higher number of genes, thus reducing the likelihood of selection acting on the same genes in multiple species or locations, as it has been argued in the case of osmoregulation in threespine sticklebacks [7,8]. Similarly, partial parallel patterns of genetic differentiation have been observed between two whitefish sympatric species pairs (a normal benthic and a dwarf limnetic) across lakes, suggestive of polygenic adaptation [53-55].

## Conclusions

The distinct signatures of selection between North American eels could be attributable to differences in larval migration between species. Alternatively, the fact that many genes of small effect likely shape adaptive pathways (i.e. metabolism, growth, osmoregulation, pathogen resistance) could explain the private signatures of spatially varying selection with no shared genetic-by-environment associations between European and American eel. As an alternative to candidate loci approaches, high-density genome-wide scans using next-generation sequencing and genotyping-by-sequencing approaches [7,56] might be more adequate. A recent study using RAD (Restriction site Associated DNA) sequencing generated a SNP resource for European eel consisting of 82,425 loci and over 375,000 SNPs [57] that provides a valuable tool for future studies on parallel selection in both North Atlantic eels on a genome-wide scale.

## Methods

### Ethical statement

No experiments were conducted on the animals and animal manipulation was limited to sacrificing fish, using the least painful method to obtain tissue samples for DNA extraction. In all cases, in order to minimize the suffering of the animals used in the study, fish were deeply anaesthetized with MS-222 (3-amonobenzoic acid ethyl ester) or 2-phenoxyethanol 1% and then painlessly sacrificed. All procedures were conducted by technical staff, who had all the necessary fishing and animal ethics permits (please see in the Additional file 1: Appendix 1).

### Sampling

**Figure 2:**
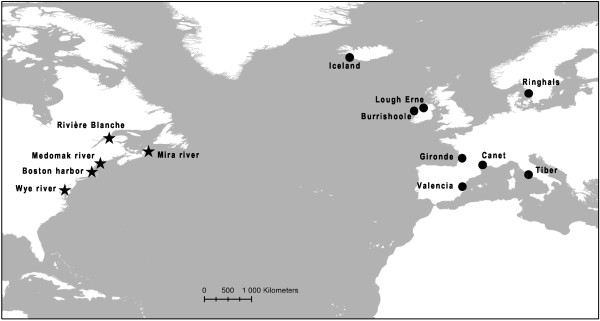
Sampling locations of European eel (circles) and American eel (stars) individuals used for comparison.

A total of 321 European eel (*Anguilla anguilla*) individuals were collected at 8 locations across the geographical distribution of the species, from Iceland to the Mediterranean Sea (Table 4; Figure 2). All individuals were glass eels caught by electrofishing (Iceland) and fyke nets (remaining localities). Individuals from Iceland were collected at four separate sampling sites in southwestern Iceland, but pooled to increase sample size. Additionally, 20 American eel (*Anguilla rostrata*) individuals collected at Rivière Blanche (Québec, Canada), Mira River (Nova Scotia, Canada), Wye River (MD, US), Medomak River (ME, US) and Boston Harbor (MA, US) were used for comparison. Genomic DNA was extracted using standard phenol-chloroform extraction.

**Table 4:** Sampling details including number of individuals per sampling location (N), latitude, longitude and sea-surface temperature at river mouth averaged across the 30 days preceding the sampling date

**Country**	**Location**	**N**	**Latitude**	**Longitude**	**Date**	**Temp (°C)**
Spain	Valencia	44	39°46′ N	0°24′ W	15 January 2010	15.05
Italy	Tiber	39	41°73′ N	12°23′ E	30 December 2007	14.17
South France	Canet	40	42°70′ N	3°15′ E	23 January 2008	13.24
West France	Gironde	40	44°86′ N	0°42′ W	16 April 2008	11.26
Ireland	Burrishoole	39	53°90′ N	9°58′ W	14 March 2005	9.57
Northern Ireland	Lough Erne	39	54°46′ N	7°77′ W	1 July 2008	13.85
Sweden	Ringhals	40	57°21′ N	12°27′ E	15 March 2008	4.19
Iceland		40			May – June 2001	8.45
	Stokkseyri	10	63°81′ N	21°04′ W		
	Vifilsstadvatn	10	64°07′ N	21°87′ W		
	Seljar	10	64°56′ N	22°31′ W		
	Vogslækur	10	64°69′ N	22°33′ W		

### SNP genotyping

We examined the panel of 100 coding-gene SNPs developed by Gagnaire et al. [26] in American eel. 20 out of the 100 primer sets did not give good amplification products in European eel and were excluded. All individuals were genotyped at 80 coding-gene SNPs: 47 SNPs that were detected as outliers between samples from Florida and Québec using RNA-sequencing data (including 4 SNPs identified within allozyme-coding genes showing clinal variation in Williams et al. [33]) and 33 SNPs that were not outliers (Table 2). SNP genotyping was conducted using the Kbioscience Competitive Allele-Specific PCR genotyping system (KASPar) (Kbioscience, Hoddeston, UK).

### Data analysis

Allele frequencies, measures of genetic diversity including polymorphism at the 95% (P_95_) and 99% level (P_99_), observed (H_o_) and expected (H_e_) heterozygosities and deviations from Hardy-Weinberg equilibrium were calculated using GENEPOP [58]. In all cases, significance levels were corrected for multiple comparisons using the sequential Bonferroni technique [59].

Overall genetic differentiation (F_ST_) was calculated in GENEPOP. Population structure was further investigated using STRUCTURE v.2.3.4 [60], which also allowed us to test the presence of hybrids in the data set. We assumed an admixture model, uncorrelated allele frequencies and we did not use population priors. Given that two panmictic species were analyzed, we assumed *k* = 2 and conducted 10 replicates to check the consistency of results. A burn-in length of 100,000 steps followed by one million additional iterations was performed.

We used two different approaches to test for evidence of local selection. First, we searched for elevated population differentiation using F_ST_-based outlier analyses. We used the selection detection workbench LOSITAN [61], which uses a coalescent-based simulation approach to identify outliers based on the distributions of heterozygosity and F_ST_[62]. First, LOSITAN was run using all SNPs to estimate the mean neutral F_ST_ as recommended by Antao et al. [61]. After the first run, the mean neutral F_ST_ was re-computed by removing those SNPs outside the confidence interval in order to obtain a better approximation of the mean neutral F_ST_. This mean was then used to conduct a second and final run of LOSITAN using all SNPs. The analysis was performed on the whole data set divived according to sampling location. An estimate of p value was obtained for each SNP. We used a threshold of 0.95 and a false discovery rate of 0.1 to minimize the number of false positives.

Outlier SNPs were also detected using BAYESCAN [63], a Bayesian method based on a logistic regression model that separates locus-specific effects of selection from population-specific effects of demography. Outlier analysis was conducted on the whole data set divided according to sampling location. BAYESCAN runs were implemented using default values for all parameters, including a total of 100,000 iterations after an initial burn-in of 50,000 steps. Posterior probabilities, q values and alpha coefficients (positive values indicate diversifying selection, negative values are indicative of balancing selection) were calculated. A q-value of 10% was used for significance.

As an alternative to F_ST_-outlier tests, our second approach for identifying targets of local selection was to test for significant statistical associations between allelic frequencies and environmental variables following Gagnaire et al. [26], using a generalized linear model in r. Environmental variables used included degrees North latitude, degrees East/West longitude, and sea-surface temperature at river mouth averaged across the 30 days preceding the sampling date. Sea-surface temperature data were retrieved from the IRI (International Research Institute for Climate and Society) Climate Data Library (http://iridl.ldeo.columbia.edu/) database “NOAA NCDC OISST version2 AVHRR SST: Daily Sea Surface Temperature”. We also searched for SNP-environment associations using BAYENV [38], which tests for covariance between candidate SNP allele frequencies and environment variables that exceed the expected covariances under genetic drift. First, SNP frequencies at all loci were used to describe how allele frequencies covary across populations, hence avoiding population-specific effects of demography (even though a panmixia scenario is most likely to apply for European eel). After the covariance matrix was estimated, the program determined the Bayes factors for the environmental variables of interest. Bayes Factors >3 were considered indicative of an allele frequency correlation with an environmental variable.

## Availability of supporting data

The data set supporting the results of this article is available from Dryad: http://datadryad.org/resource/doi:10.5061/dryad.jn800/1.

## Competing interests

The authors declare no competing interest.

## Authors’ contributions

MMH and LB conceived and designed the project. MGU and JMP conducted population genetics analyses with help from MMH, TDA, ALF and MWJ. MGU and JMP wrote the manuscript with contributions from MMH, LB, PAP, ALF, MWJ, TDA, JF, PKB and BJ. All authors read and approved the final version of the manuscript.

## Supplementary Material

Additional file 1: Appendix 1 Supplementary ethical statement. Click here for file
