# Supplementary material for: Do North Atlantic eels show parallel patterns of spatially varying selection?
Source: BMC Evol Biol. 2014 Jun 20;14:138. doi: 10.1186/1471-2148-14-138 (PMC4069275; doi:10.1186/1471-2148-14-138)
Supplement: Additional file 1: Appendix 1 — Supplementary ethical statement. [file 1471-2148-14-138-S1.docx]

**Additional File 1.**

**Appendix 1.** Supplementary Ethical Statement.

In the case of Icelandic samples, the research was approved by Holar University College Ethical Committee and conducted according to guidelines and laws on animal welfare in Iceland. Regarding samples from Ireland, the Burrishoole river is the national (Government of Ireland) index river for research into Atlantic salmon and European eel and all sampling was carried out under authorisation (Sec. 4) of the Fisheries Act 1959-2003 by permission of the Department of Agriculture, Food and Marine. Spanish samples were obtained from professional fishermen with sampling and ethical treatment of animals approved by the Consejeria de Medio Ambiente of the Comunidad Autonoma de Valencia, which is the legal authority operating in the region. Italian samples were obtained from commercial catches, under the supervision of the University of Rome Tor Vergata, which is the operating unit dealing with eel monitoring and sampling in Italy. Eleonora Ciccotti is the responsible of the unit and also coordinator of the actions of the Eel Management Plan (CE 1100/2007) for the Ministry of Agricultural, Food and Forestry Policies, General Directorate of Fisheries and Aquaculture. The authorization of the Ethics Committee of the University of Rome Tor Vergata was not required since the fishery data collection programs deals with commercial catches. In regards to samples from France, authorization to collect samples was given by the Ministry of Agriculture and Fisheries, with an “autorisation d’expérimenter” provided by the National Ethic Committee. Samples from Sweden were obtained by the Coastal Institute of the Swedish Board of Fisheries. The permit (C 43/11) was approved by the Swedish Ethical Committee on Animal Research. Finally, American eel samples were obtained in accordance with the Guide for the Care and Use of Laboratory Animals of the Canadian Council on Animal Care in Science. The protocol was approved by the Committee on the Good Care of Animal Experiments of Université Laval.
